# The effect of manual therapy and exercise on age-related lung function: study protocol for a randomised controlled trial

**DOI:** 10.1186/s13063-019-3257-z

**Published:** 2019-03-13

**Authors:** Roger Engel, Sandra Grace, Suzanne Broadbent

**Affiliations:** 1 Department of Chiropractic, Macquarie University, North Ryde, Sydney, NSW 2109 Australia; 2 School of Health and Human Sciences, Southern Cross University, Lismore, Australia; 3 School of Health and Sports Sciences, University of the Sunshine Coast, Sippy Downs, Australia

**Keywords:** Ageing, Manual therapy, Spinal mobilisation, Spinal manipulation, Exercise, Randomised controlled trial, Lung function, Trial protocol

## Abstract

**Background:**

Ageing is associated with a range of anatomical and physiological changes. Establishing whether a change is part of ‘normal’ ageing or the early signs of disease will affect management strategies. Progressive stiffening of the thoracic spine, decreasing chest wall compliance and declining lung function begin as early as 40 years of age. Administering an intervention such as manual therapy, which has the potential to mitigate age-related changes in the thoracic spine and chest wall, has the potential to improve thoracic compliance and lung function. The aims of this trial are to investigate whether manual therapy can mitigate the effects of age-related changes in lung function and whether there is a difference in effect between different forms of manual therapy.

**Methods:**

The study design is a randomised controlled trial of 372 people with no history of respiratory disease between the ages of 50 and 65 years. The cohort will be divided into three equal groups. The first group will receive a simple 10-min treadmill walking program (Ex). The second group will receive joint mobilisation (MB) of the thoracic spine and ribs plus the same walking program (MB + Ex). The third group will receive joint manipulation (MT) of the thoracic spine and ribs plus the same walking program (MT + Ex). All interventions will be administered a total of six times over a 3-week period. The primary outcome measure is lung function: forced expiratory volume in the 1st second and forced vital capacity. The secondary outcome measures include chest wall expansion (tape measurements) and quality of life measurements (36-Item Short Form Health Survey). Outcome measurements will be taken by blinded assessors on four occasions over a 9-week period. Adverse event data will be gathered at the beginning of each intervention session.

**Discussion:**

This randomised controlled trial is designed to investigate whether manual therapy can mitigate the effects of age-related changes in lung function and whether there is a difference in effect between different forms of manual therapy. This is the first fully powered trial designed to test this hypothesis on healthy males and females in this age range.

**Trial registration:**

Australian New Zealand Clinical Trials Registry (ANZCTR), 12616001317482. Registered on 20 September 2016.

**Electronic supplementary material:**

The online version of this article (10.1186/s13063-019-3257-z) contains supplementary material, which is available to authorized users.

## Background

Ageing is associated with a range of anatomical and physiological changes involving the cardiovascular, pulmonary and musculoskeletal systems [1–3]. Establishing whether a change is part of ‘normal’ ageing or the early signs of disease is, however, often difficult. In the case of the musculoskeletal system, ‘normal’ age-related changes in the thoracic spine and chest wall include a reduction in spinal muscle thickness, costal cartilage calcification, osteoporosis and spondylosis [4–6]. These changes start to manifest as early as 40 years of age and can lead to progressive stiffening of the spine, decreasing chest wall compliance and declining lung function [5, 7].

As chest wall compliance is a major determinant of static lung volumes, improving compliance leads to improvements in lung volumes. Lung volume is usually assessed using functional lung assessments such as forced vital capacity (FVC) and forced expiratory volume in the 1st second (FEV_1_).

If an intervention designed to improve chest wall compliance was administered before the age-related musculoskeletal changes in the thoracic spine and chest wall became irreversible, it could mitigate those changes and therefore have an impact on lung function [4–7].

Sandoz was the first to test this concept in 1976 when he proposed that altering the ’dynamic capabilities’ of the thoracic kyphosis would improve lung function [8]. In his seminal work, Sandoz administered a 6-week course of manual therapy (mobilisation or manipulation) to the thoracic spines of 20 women between the ages of 60 and 65. He found that both interventions produced clinically meaningful increases in lung function with manipulation producing a greater increase than mobilisation. Interestingly, he noted that the maximum improvement in lung function was reached after 3 weeks of either intervention, with no additional improvements achieved following a further 3 weeks of manual therapy. This means that there may be a functional limit to the effect that manual therapy can have on the reversibility of age-related declining lung function.

Others have repeated this experiment on younger people (18–28 years) with similar results [9–11]. However, those studies were conducted on small sample sizes and did not address Sandoz’s central issue of the reversibility of age-related lung function. This is the first fully powered trial designed to test this concept on healthy older people.

The aims of this trial are to investigate whether manual therapy can mitigate the effects of age-related changes in lung function and whether there is a difference in effect between two different forms of manual therapy.

## Methods/design

### Study design

The trial is designed as a randomised controlled trial and fulfils the requirements of the Standard Protocol Items: Recommendations for Interventional Trials (SPIRIT) checklist [12] (Additional file 1). The trial will evaluate 372 participants randomly allocated to three equal groups. Participants in Group 1 (Ex), the active control, will undertake a simple exercise program involving a standardised 10-min walking program performed on a treadmill. Participants in Group 2 (MB + Ex) will have manual therapy in the form of joint mobilisation (MB) administered to their thoracic spine and ribs prior to performing the same exercise regime as Group 1. Mobilisation consists of a pre-determined series of manoeuvres (mobilisation protocol) designed to increase joint mobility in the thoracic spine and rib cage (Grade III–IV mobilisation as per Maitland [13]). Participants in Group 3 (MT + Ex) will have manual therapy in the form of joint manipulation (manipulation protocol) administered to their thoracic spine and ribs prior to performing the same exercise regime as Group 1. Manipulation (MT) consists of two separate manipulations (Grade V mobilisation as per Maitland [13]), each involving a high velocity low amplitude (HVLA) thrust directed at the inter-vertebral, costo-vertebral and costo-transverse joints. The first manipulation will be delivered at the level of the upper/middle thoracic spine, while the second will be at the level of the middle/lower thoracic spine. Two different manipulation techniques are available under the trial’s manipulation protocol: supine and sitting. The supine technique, referred to as the ‘Opposite-side thenar/transverse drop’ [14] or ‘Supine wing’ [15] technique, can be delivered with the participant’s thoracic spine in flexion or extension. The sitting technique, referred to as a ‘Long-axis distraction’ [14] or ‘Sitting winglift’ [15] technique, can be delivered using an axilla or elbow hold. The choice of technique will be determined by the treating clinician based on biomechanical factors such as the patient-to-practitioner size ratio. All MT intervention will be administered as non-specific, multi-joint (group) manipulations. Administering MT in this way reduces the total number of manipulations required to address the thoracic spine within a single intervention session, as each manipulation has the potential to affect several thoracic vertebrae and their associated ribs simultaneously. All Ex will be performed immediately after MB and MT intervention, as this has been shown to enhance the synergistic effect of combining the two interventions [9, 16–18]. All intervention sessions will last between 15 and 20 min.

Participants will be recruited through self-referral in response to public advertisements placed in the local printed media and articles broadcast on the radio and/or television calling for volunteers for the trial. For logistical reasons the trial will be conducted at two sites: Southern Cross University’s Health Clinic in Lismore and Southern Cross University’s Health Clinic in the Gold Coast. The two sites will run sequentially with enrolment at the Gold Coast site not commencing until completion of the trial at the Lismore site. The main reasons for conducting the trial in this manner are recruitment and quality assurance. As Lismore is a medium-sized town in rural New South Wales (population 27,000), it is unlikely that the Lismore site will be able to generate sufficient participants for full enrolment. Adding the Gold Coast site, with its higher proportion of people over the age of 50, will improve the possibility of full enrolment. As the two sites are located approximately 98 km apart, achieving an acceptable level of quality assurance with the resources available mandates that the two sites be run consecutively.

### Outcome measurements

The primary outcome measure is lung function: FEV_1_ and FVC. The secondary outcome measures include chest wall expansion (tape measurements) and quality of life measurements (36-Item Short Form Health Survey, SF-36). The metric for these measures is mean change from baseline. All outcome measurements will be taken by blinded assessors on four occasions over a 9-week period: at baseline and then at weeks 3, 6 and 9.

### Participant characteristics

The trial includes both males and females between the ages of 50 and 65 years with no history of respiratory disease. Participants will be excluded if they have a history of respiratory disease, are currently smoking, cannot walk unaided and unassisted, have an existing diagnosis of osteoporosis or thoracic joint instability, present with acute pain on thoracic joint range of motion testing, have below normal chest wall musculature for their age and gender, exhibit a high level of anxiety related to receiving thoracic MB or MT or are contra-indicated for MB or MT as determined by a physical screening examination. Inclusion and exclusion criteria are described in Table 1.

**Table 1:** Inclusion and exclusion criteria for participation

Inclusion criteria	Exclusion criteria
• Male and female • 50–65 years • No history of respiratory disease • Currently non-smoking (> 6 months) • Willingness to provide written consent • Willingness to participate in and comply with study requirements	• Inability to walk unaided and unassisted • Existing diagnosis of osteoporosis or thoracic joint instability • Present with: o Acute pain on thoracic joint range of motion testing o Below normal chest wall musculature o High level of anxiety related to receiving thoracic manual therapy o Contra-indicated for thoracic mobilisation and/or manipulation (physical screening examination) • Inability to understand English • Inability to provide informed consent, e.g. people with a cognitive impairment, intellectual disability or mental illness

### Protocol description

**Fig. 1:**
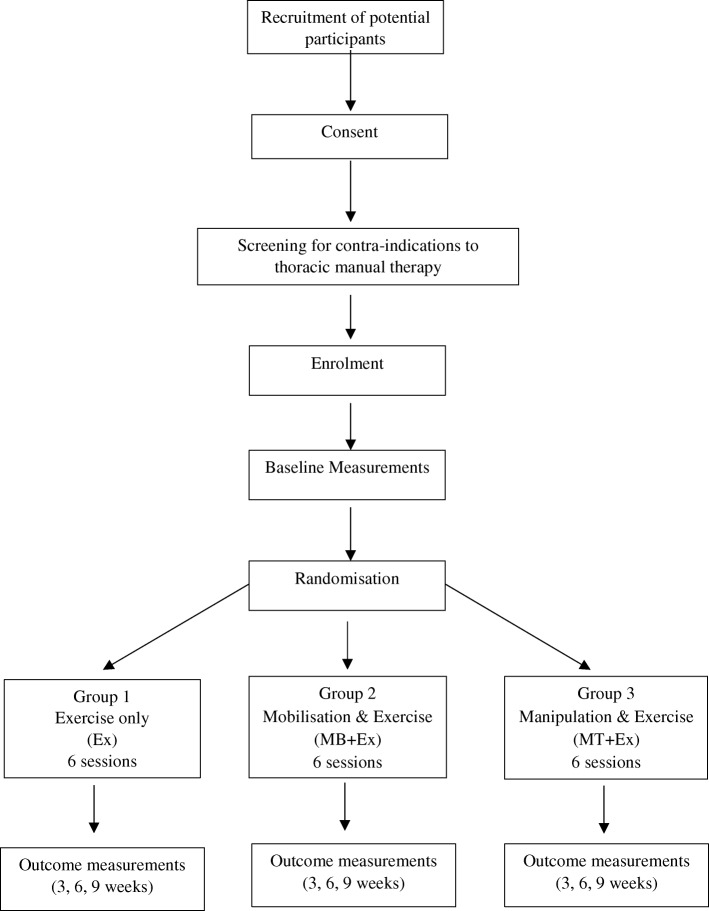
Flow of participants through the trial. *Ex* exercise, *MB* mobilisation, *MT* manipulation

Volunteers who meet the inclusion criteria will be given an information sheet and asked to provide written consent to participate in the trial. They will then undergo a physical examination screening for the presence of other contra-indications to thoracic MB or MT such as thoracic spine surgery or recent trauma followed by an information session designed to familiarise them with the use of a treadmill. After a volunteer has successfully completed these tasks, baseline measurements will be taken. These include age, height, weight, blood pressure, heart rate, lung function assessment (spirometry), chest wall expansion (tape measure) and quality of life scores (SF-36). A participant is then enrolled in the trial, given a trial-specific identification (ID) number and allocated to a group on a sequential basis using a computer-generated random sequence list drawn up prior to the start of the trial by a person not involved in any other aspect of the trial. Participants will be directed to not reveal their group allocation to the blinded assessor during assessment. The study protocol flow of participants is outlined in Fig. 1.

**Fig. 2:**
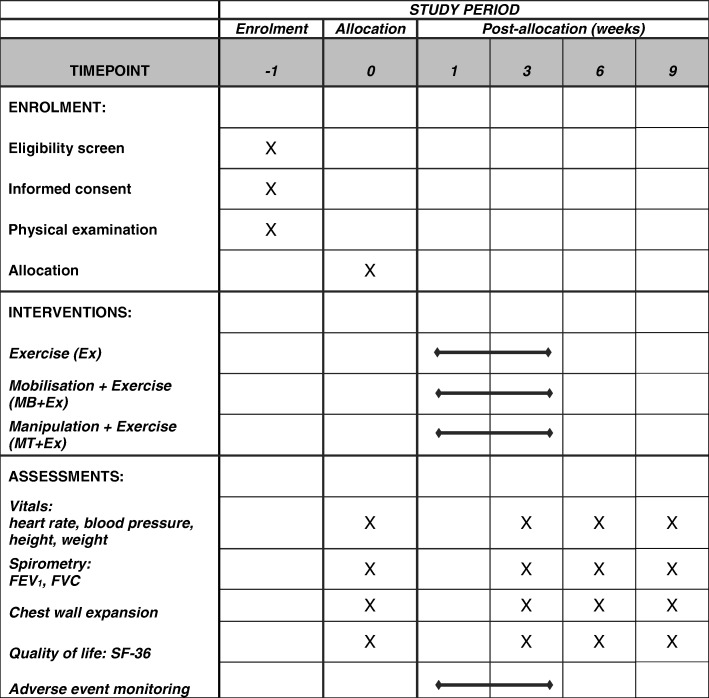
SPIRIT figure showing times points for enrolment, interventions and assessments. *Ex* exercise, *MB* mobilisation, *MT* manipulation, *FEV*_*1*_ forced expiratory volume in the 1st second, *FVC* forced vital capacity, *SF-36* 36-Item Short Form Health Survey

All outcome measures will be taken by an assessor who is blinded to a participant’s group allocation. Lung function measurements (spirometry) will be measured in the standing position and include FEV_1_ and FVC. The initial treadmill walking speed will have been determined at the information session conducted in conjunction with the physical screening examination. This speed is set at a participant’s ‘comfortable walking pace’ as agreed to at that screening session. The SPIRIT figure showing the respective time points for enrolment, interventions and assessments is provided as Fig. 2.

All MB and MT will be administered by students enrolled in the Masters of Osteopathic Medicine program at Southern Cross University who have been trained in the relevant manual therapy techniques used in this trial. They will be under supervision at all times by a registered osteopath.

A manipulation will be deemed successful by the presence of an audible release. All releases will be recorded on the participant’s file by the treating intern as either ‘absent’ (0), ‘single’ (1), or ‘multiple’ (2) for each manipulation. Should a deviation from the intervention protocol occur, the supervisor will be on hand to either approve the deviation or ensure a return to protocol. Any protocol violations, whether for clinical or non-clinical reasons, will be recorded on the participant’s file by the treating intern at each intervention session.

### Consent process

The consent process followed in this trial will be the same for all participants. Potential participants will receive the patient information and consent form (PICF) explaining the trial’s processes and procedures, including consent to publish, and what is expected from them during the trial. Each participant will be given the opportunity to ask questions about their involvement in the trial and to have those questions answered by a researcher associated with the trial prior to providing consent. The trial ID number will be used when gathering and recording all information about a participant. A list containing the contact details of all participants and their corresponding ID numbers will be kept separate from all other data. All paper copy forms will be stored in a locked filing cabinet in the relevant clinical facility during the trial. Once involvement in the trial is completed (post-week 9), all data from these files will be scanned and transferred to a password protected encrypted file and stored on the university computer of principal investigator 1 (RE). These electronic files will be backed up on an external hard drive and stored in a locked filing cabinet in the university office of principal investigator 2 (SG). The trial was approved by the Human Research Ethics Committee (HREC) of Southern Cross University, approval number ECN-16-211.

### Sample size calculation

Sample size calculation was based on change in FVC and the ability to detect a difference in lung function from baseline. The minimum clinically important difference (MCID) for FVC is 200 ml with a standard deviation of 48 ml obtained from previous studies on people with and without a history of respiratory disease [9, 16, 17]. With a power of 0.8 (80%), an alpha of 0.05 and an MCID for FVC of 200 ml, the minimum sample size per group was calculated to be 112. Assuming a drop-out rate of 10%, the minimum cohort size required is 372 (three groups of 124).

### Statistical analysis

Data will be reported as group means, standard deviations and 95% confidence intervals. Analysis will be performed as an analysis of covariance (ANCOVA) for difference between groups with baseline as a covariate. Standard errors will be calculated using a non-parametric bootstrap to allow for the different error variances for each group. A *p* value of < 0.05 has been set for statistical significance. For outcomes found to be statistically significant, the proportion of participants with a change greater than the MCID will be calculated for each outcome. The number needed to treat will be calculated using Bender’s method for confidence intervals. Missing data will be accounted for by using an intention-to-treat analysis with data from subjects lost to follow-up imputed using the multiple imputation method.

### Data monitoring

The data safety monitoring board (DSMB) appointee who will oversee the study is the Osteopathy Professional Experience Learning Co-ordinator in the School of Health and Human Sciences at Southern Cross University. The DSMB appointee will meet with one or more of the principal investigators on a regular basis to review the study data. As this trial includes the administration of manual therapy by supervised interns, the meetings with the DSMB appointee will focus on safety by reviewing adverse event (AE) data as it is collected during the trial. While severe or unexpected AEs are reported to the relevant Ethics Committee (Institutional Review Board) as a matter of standard procedure in any clinical trial, the DSMB meetings in this trial will track the rate of expected AEs, i.e. the mild to moderate AEs, as a way of monitoring whether participants are being placed at ‘additional’ risk by being treated by interns.

### Adverse events

The risks of harm or discomfort to participants in this project primarily relate to the potential for AEs resulting from MT intervention. The majority of reported AEs associated with these interventions are mild (muscle soreness and local discomfort), self-limiting, require no further medical attention and resolve within 48 h [19]. Moderate AEs, such as fracture, have also been reported following spinal manipulation and have been estimated to occur at a rate of 1 in 40,000 [20]. Reports of major or catastrophic AEs resulting from spinal manipulation have appeared in the literature with nearly all associated with neck (cervical) manipulation. This trial involves manipulation of the thoracic spine and ribs only and does not include any neck manipulation. The MT techniques used in this trial are similar to the ones used in three previous studies on people with chronic obstructive pulmonary disease (COPD) [16, 17, 21]. There were no major or moderate AEs reported in those studies. Mild AEs associated with MT intervention were reported at a rate of 15% (18 out of 112) in the study on patients with moderate COPD (average age 56.1 years) [16], at a rate of less than 1% (2 out of 403) in the study on patients with moderate to severe COPD (average age 65.5 years) [17] and at a rate of 29% (21 out of 72) in the case series on patients with moderate to severe COPD (average age 79.1 years) [21]. As the trial reported here is being conducted on healthy participants with no history of respiratory disease, it is reasonable to predict that the risk of harm or discomfort to participants will be less than that in the previous studies on people of a similar age with respiratory disease. However, as this trial includes the administration of manual therapy by clinicians with limited clinical experience (interns), we have used an incident rate of 41% for mild AEs, taken from Carnes et al. [19], as the threshold for triggering an automatic halt to the study.

## Discussion

Ageing is characterised by changes in the thoracic spine and chest wall. These changes result in a decrease in chest wall compliance that is associated with declining lung function. Improving compliance carries with it the potential for increasing lung function. Administering manual therapy in an attempt to improve chest wall compliance is not a new concept. However, previous studies have only administered the intervention for this reason to young healthy cohorts with and without the presence of identified restricted thoracic joint mobility [9–11, 22, 23] or to older people with chronic diseases that directly impact lung function such as ankylosing spondylitis [24–26] or COPD [16–18, 27–31]. One study has investigated the effect of manual therapy on healthy, older individuals, but that study was not adequately powered, only involved women and did not include a control group for comparison [8].

The importance of being able to mitigate the age-related decrease in chest wall compliance lies in the flow-on effect it has on lung function and the ability to maintain activity levels as a person ages. With physical inactivity now seen as a public health priority globally, coordinated change is required at a social, cultural, environmental and policy level to reverse the growing trend towards inactivity [32, 33]. Improving chest wall compliance in order to facilitate an increase in activity levels in older people is one approach to achieving this. Using lung function as an outcome measure is a simple, cost-effective and reliable way to measure the effectiveness of this approach.

We hypothesise that administering manual therapy to improve chest wall compliance will ultimately help to maintain activity levels as people age by mitigating one of the drivers of inactivity, declining lung function. The fully powered randomised controlled trial being reported in this manuscript will achieve this by examining the effect of manual therapy on lung function in 372 healthy people between the ages of 50 and 65 years.

## 3.1. Trial status

Recruitment for the trial is ongoing at the time of submission.

## Additional file

Additional file 1: Standard Protocol Items: Recommendations for Interventional Trials (SPIRIT) 2013 checklist. Recommended items to address in a clinical trial protocol and related documents. (DOC 120 kb)

## Abbreviations

AE: Adverse event
ANCOVA: Analysis of covariates
COPD: Chronic obstructive pulmonary disease
DSMB: Data safety monitoring board
Ex: Exercise
FEV_1_: Forced expiratory volume in the 1st second
FVC: Forced vital capacity
HREC: Human Research Ethics Committee
HVLA: High velocity low amplitude
ID: Identification
MB: Mobilisation
MCID: Minimum clinically important difference
MT: Manipulation
PICF: Patient information and consent form
SPIRIT: Standard Protocol Items: Recommendations for Interventional Trials
